# Kawasaki disease complicated with shock syndrome and macrophage activation syndrome in children: a case report

**DOI:** 10.3389/fped.2025.1675460

**Published:** 2025-10-16

**Authors:** Juxia Shu, Jingwei Sun, Jiachen Li, Mengjin Wang, Nan Dong, Dong Qi, Fang Liu

**Affiliations:** ^1^ Pediatric Department, Bengbu First People’s Hospital, Bengbu, Anhui, China; ^2^ Department of Ultrasound, Bengbu First People’s Hospital, Bengbu, Anhui, China; ^3^ Department of Radiology, Bengbu First People’s Hospital, Bengbu, Anhui, China; ^4^ Pediatric Heart Center, Children’s Hospital of Fudan University, Shanghai, China

**Keywords:** Kawasaki disease, Kawasaki disease shock syndrome, macrophage activation syndrome, case report, giant coronary artery aneurysm

## Abstract

Kawasaki disease (KD) is an acute self-limited vasculitis of unknown etiology. Clinically, a small proportion of patients may develop shock syndrome or macrophage activation syndrome (MAS); however, KD shock syndrome (KDSS) complicated by MAS is extremely rare. This article provides a detailed report on the clinical diagnosis and treatment course of a child with KD complicated by KDSS and MAS.

## Introduction

Kawasaki disease (KD) is an acute febrile systemic vasculitis of unknown etiology, commonly affecting children under the age of 5. It often involves medium-sized arteries, especially the coronary arteries. Among untreated children, 15%–25% may develop coronary artery abnormalities (1). KD shock syndrome (KDSS) is a severe type of KD, defined by hypotension and shock during the acute phase of KD, requiring fluid resuscitation or vasoactive drugs, with an incidence rate of 1%–7% (2, 3). Macrophage activation syndrome (MAS) is a rarer and more severe complication of KD, classified as a special type of secondary hemophagocytic lymphohistiocytosis (HLH). MAS is more common in children with KD over the age of 5, with an incidence rate of 1.1%–1.9% (4). Reports of KDSS combined with MAS are extremely rare. Here, we report a case of KD complicated by KDSS, MAS, severe pneumonia, acute cholecystitis, and a giant coronary artery aneurysm (CAA).

## Report of the case

This case report study was approved by the Medical Research Review Board of Bengbu First People's Hospital.

A 7-year-old boy weighing 28 kg was admitted to a local hospital for “fever with cough for 6 days.” During his hospitalization, laboratory tests showed the following results: white blood cell (WBC) 2.49 × 10^9^/L (neutrophils 91.3%), hemoglobin (Hb) 128 g/L, platelet (PLT) 106 × 10^9^/L, C-reactive protein (CRP) 110.94 mg/L, serum amyloid A (SAA) 156 mg/L, interleukin-6 (IL-6) 104 pg/mL, procalcitonin (PCT) 0.69 ng/mL, albumin 37.35 g/L, alanine aminotransferase (ALT) 119 U/L, and aspartate aminotransferase (AST) 106 U/L. Ultrasound of the appendix and mesenteric lymph nodes showed no significant abnormalities, while chest x-ray revealed patchy areas of increased density in the left lung. After 1 day of intravenous azithromycin and ceftriaxone, the child developed a diffuse rash, accompanied by abdominal pain, primarily in the upper abdomen. Oral “cetirizine” had no effect. He was referred to our hospital on the 7th day of his illness.

**Figure 1:**
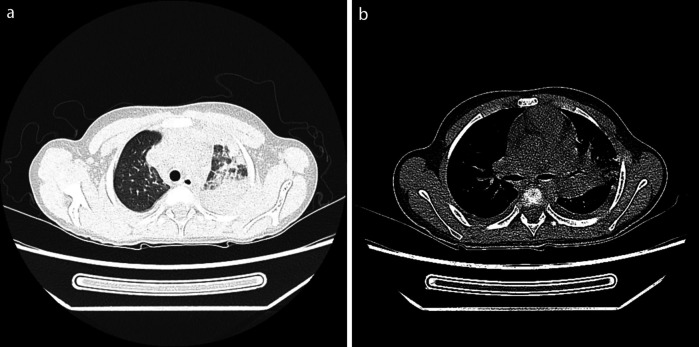
Chest CT. Left lobar pneumonia **(a)** and pleural effusion **(b)**.

**Figure 2:**
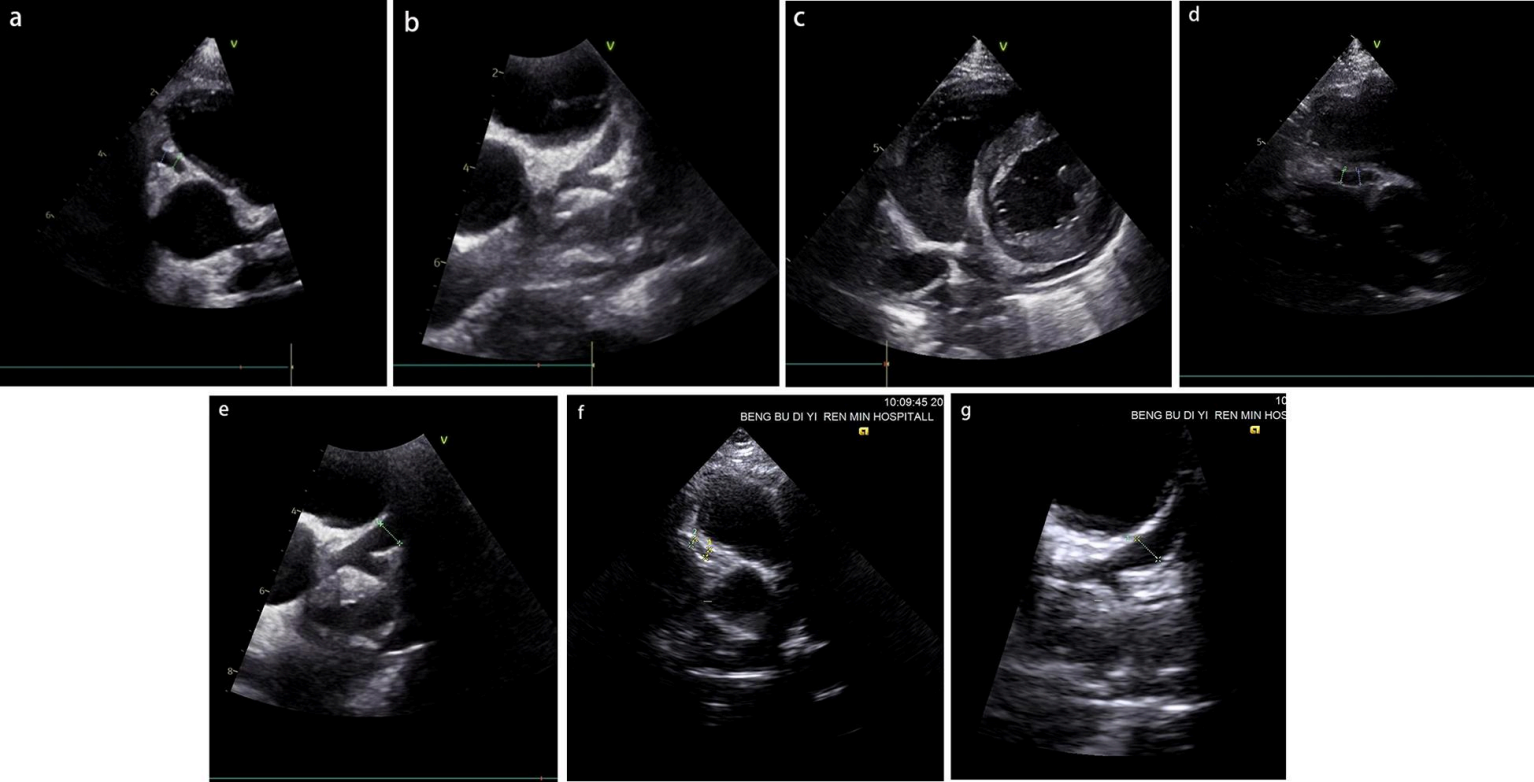
**(a–c)** The results of the patient's initial coronary artery echocardiography following hospital admission (on day 12 of illness). **(d,e)** The findings obtained prior to discharge (on day 33 of illness). **(f,g)** The results from the most recent follow-up evaluation (6 months after the onset of illness). RCA dilation with a diameter of 3.5 mm **(a)**, left coronary artery dilation with LMCA 4.0 mm, left ascending artery (LAD) 4.4 mm, LCX 3.2 mm **(b)**, and mild pericardial effusion **(c)** were shown. RCA dilation with a diameter of 4.3 mm **(d)**, left coronary artery dilation with LMCA 3.7 mm, LAD 6.3 mm, LCX 4.3 mm **(e)**, RCA dilation with a diameter of 2.7 mm **(f)**, left coronary artery dilation with LMCA 3.2 mm, LAD 5.0 mm, LCX 2.6 mm **(g).**

**Figure 3:**
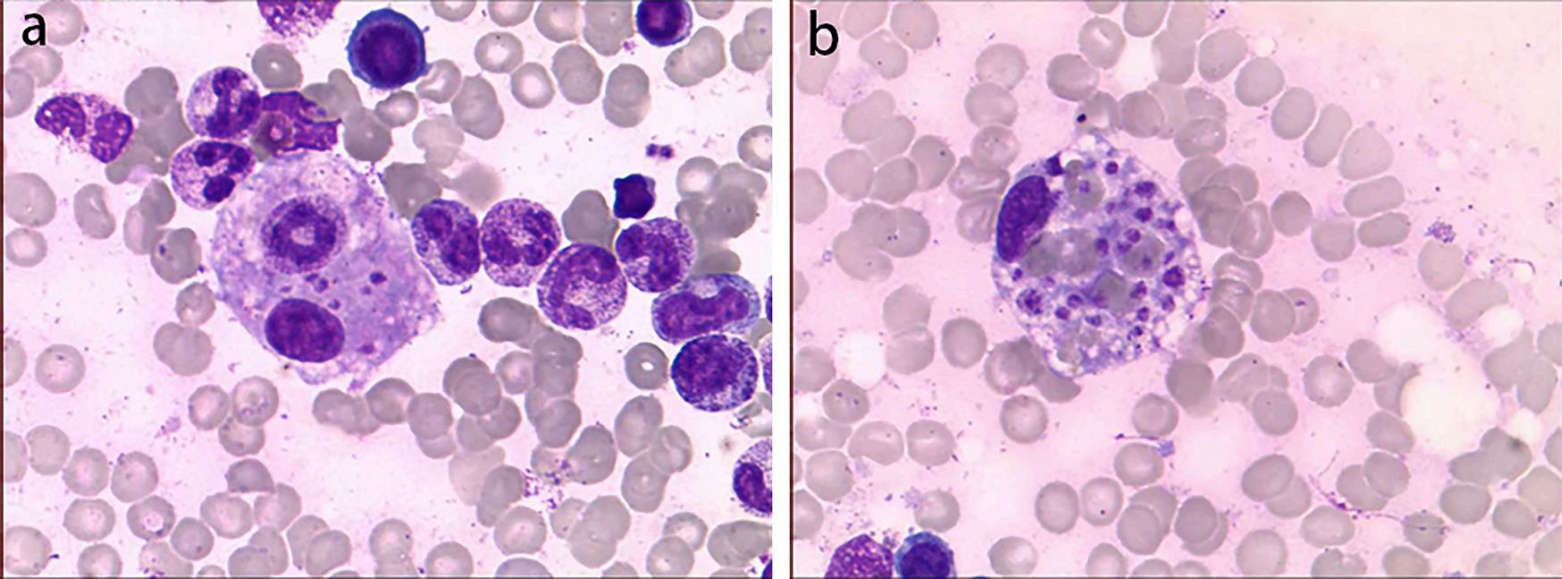
Bone marrow biopsy smear. Hemophagocytic cells were shown. The left image **(a)** shows phagocytic granulocytes, and the right image **(b)** shows platelets being phagocytized.

**Figure 4:**
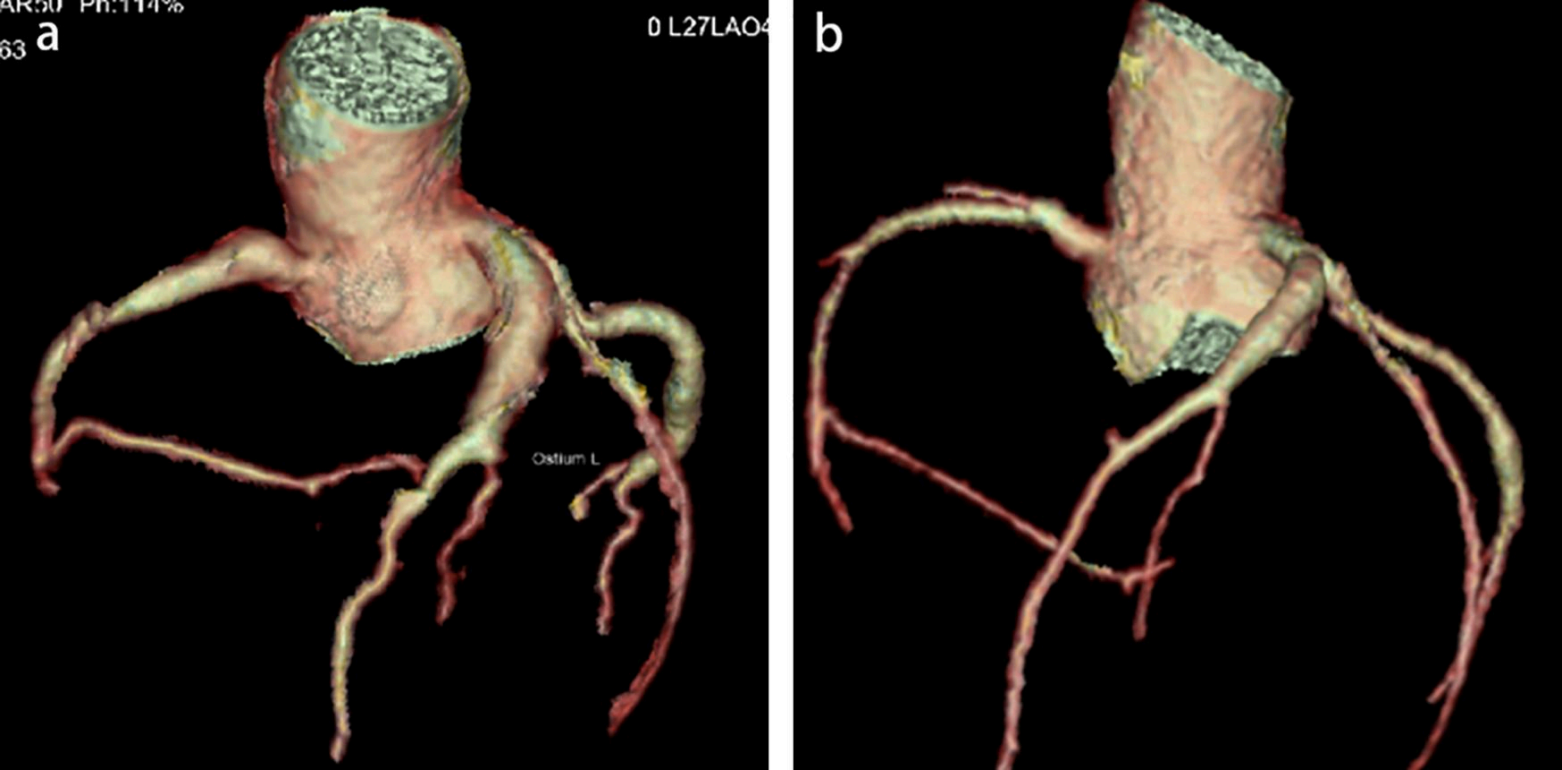
Coronary artery computed tomography angiography (CTA, 3 months after the onset of illness). CTA before discharge **(a)** shows long segment aneurysms in RCA, LAD, and LCX, respectively, with a maximum diameter of 6.8 mm in LAD. CTA at 3 months follow-up **(b)** shows significant improvement in every three branches, with a maximum diameter of 4.8 mm in LAD.

Physical examination upon admission: Temperature 38.4°C, no obvious conjunctival congestion, no dryness or redness of the lips, no “strawberry tongue,” no changes in extremities, several enlarged lymph nodes palpable in the neck. Scattered red rashes were observed over the whole body, raised above the skin surface, non-blanching on pressure, partially confluent, and without significant pruritus. Dry and moist rales were heard in both lungs. Abdomen slightly distended, with tenderness in the upper abdomen but no rebound tenderness. The liver is approximately 2 cm below the costal margin, and the spleen is approximately 1.5 cm below the costal margin. Blood pressure is significantly reduced (71/45 mmHg). Laboratory examinations: WBC count normal (4.79 × 10^9^/L), CRP elevated (158.14 mg/L), erythrocyte sedimentation rate (ESR) slightly elevated (21 mm/h), PLT count reduced (44.00 × 10^9^/L), Hb reduced (104 g/ L), SAA elevated (321.48 mg/L), IL-6 elevated (143 pg/mL), D-dimer elevated (7.23 µg/mL), albumin reduced (26.00 g/ L), transaminases elevated (ALT 136 U/L, AST 120 U/L), and pro-brain natriuretic peptide (pro-BNP) elevated (6,968 pg/mL). Chest computed tomography (CT) suggested pneumonia with pleural effusion (Figure 1), and chest ultrasound showed left-sided pleural effusion of 51 mm. Abdominal CT revealed peritoneal effusion with cholecystitis, and ultrasound showed a thickened gallbladder wall and splenomegaly. Based on the child's clinical symptoms and auxiliary examination results, the preliminary diagnosis was severe pneumonia complicated by septic shock. Methylprednisolone was administered for anti-inflammatory treatment, meropenem for infection, low molecular weight heparin for anticoagulation, fluid resuscitation, dopamine and dobutamine for blood pressure support [although foreign literature had already reported that adrenaline and noradrenaline were the firstline inotropic agents for septic shock, the guidelines in China were not updated until 2025 (5)], albumin administration for hypoalbuminemia, and intravenous immunoglobulin (IVIG, 1 g/kg/day for 2 days) for immunomodulation. After 5 days of treatment (on day 12 of illness), the child still presented with recurring fever and abdominal pain, but the rash had gradually subsided. Physical examination revealed slight conjunctival congestion, slightly red lips, and a strawberry tongue. Several enlarged lymph nodes were palpable in the neck. Blood tests indicated elevated serum ferritin levels (2,086 ng/mL) with nagetive blood culture report, so KD was highly suspected, and further tests were conducted. Neck ultrasound showed enlarged cervical lymph nodes (2 cm × 2 cm), and cardiac echocardiogram revealed left main coronary artery (LMCA) dilation (4.0 mm, *Z* score + 2.93), left circumflex artery dilation (LCX 3.2 mm, *Z* score + 3.15), left anterior descending artery dilation (LAD 4.4 mm, *Z* score + 5.73), and right coronary artery widening (RCA 3.5 mm, *Z* score + 2.36), with mild pericardial effusion (Figure 2). Hemophagocytic cells were shown on the bone marrow biopsy (Figure 3). Considering the progressive increase in ferritin levels, a sharp decrease in platelets, elevated AST, and reduced fibrinogen (from 3.52 to 1.39 g/L) during the course of illness, the child was diagnosed with KDSS, MAS, severe pneumonia, and cholecystitis. On the 12th day of illness, IVIG (2 g/kg) was administered along with a methylprednisolone pulse therapy (30 mg/kg/day for 3 days) and oral aspirin 100 mg daily for antiplatelet therapy due to his poor liver function. After finishing IVIG infusion (on day 13 of illness), the child's temperature normalized, follow-up chest CT indicated improvement of pneumonia, and corticosteroids were tapered. However, on day 20, fever recurred when methylprednisolone was tapered to 1 mg/kg/day, accompanied by mild erythema on both palms. CRP reelevated to 38.43 mg/L, suggesting an IVIG non-responding KD. Echocardiography showed a progressive CAA (Table 1). So infliximab (5 mg/kg) combined with methylprednisolone (2 mg/kg/day) was given. His fever and symptoms resolved completely, and the corticosteroid was tapered within 3 weeks. Prior to discharge (on day 33 of illness), an echocardiogram showed multiple CAAs (LMCA 3.7 mm, *Z* score + 2.24; LCX 4.3 mm, *Z* score + 4.9; left ascending artery (LAD) 6.3 mm, *Z* score + 10.49; RCA 4.3 mm, *Z* score + 4.14) (Figure 2). PLT count increased to 408 × 10^9^/L, and then oral rivaroxaban 7.5 mg and aspirin 100 mg daily were prescribed for antithrombosis (6), and coronary artery computed tomography angiography (CTA) was performed (Figure 4). The child was discharged after 30 days of hospitalization and was scheduled for regular outpatient follow-up. The CTA 3 months after discharge showed significant improvement in all three branches, with the maximum diameter 6.8 mm in LAD (Figure 4); therefore, rivaroxaban was replaced with clopidogrel. At the last follow-up (6 months after the onset of illness), the echocardiogram showed significant regression of the coronary aneurysms (LMCA 3.2 mm, *Z* score +1.08; LCX 2.6 mm, *Z* score + 1.17; LAD 5.0 mm, *Z* score + 7.24; RCA 2.7 mm, *Z* score + 0.58), and only aspirin was continued.

**Table 1:** Coronary artery diameter by echocardiography.

Coronary artery diameter	Day 12	Day 16	Day 33	Day 57	Day 104	Day 160	Day 197
LMCA (mm) *Z* score (SD)	4.0 + 2.93	3.6 + 2.01	3.7 + 2.24	3.5 + 1.77	3.2 + 1.08	3.4 + 1.54	3.2 + 1.08
LCX (mm) Z score (SD)	3.2 + 3.15	3.5 + 3.15	4.3 + 4.9	2.8 + 1.61	2.6 + 1.17	2.6 + 1.17	2.6 + 1.17
LAD (mm) *Z* score (SD)	4.4 + 5.73	4.6 + 6.23	6.3 + 10.49	5.6 + 8.74	5.4 + 8.24	5.0 + 7.24	5.0 + 7.24
RCA (mm) *Z* score (SD)	3.5 + 2.36	3.7 + 2.80	4.3 + 4.14	3.0 + 1.25	2.9 + 1.02	2.7 + 0.58	2.7 + 0.58

The coronary artery *Z* scores were calculated using the method proposed by Dallaire.

## Discussion

The diagnosis of KD primarily relies on the typical clinical manifestations. For infants and older children, symptoms may not be typical, and laboratory tests are necessary for timely diagnosis. KDSS is a severe form of KD, and children with shock onset are prone to misdiagnosis as septic shock. This patient developed a pulmonary infection followed by shock and was diagnosed with septic shock. Anti-infection and anti-shock treatment were given; both *The New England Journal of Medicine* and the Chinese expert consensus have pointed out that corticosteroids help improve severe pneumonia; therefore, we chose methylprednisolone for anti-inflammatory treatment, but the efficacy was poor (7). After reviewing the medical history, the child had a fever lasting for >5 days and no improvement with antibiotic therapy, rash, bilateral conjunctival congestion, strawberry tongue, and cervical lymph nodes, all of which met the criteria of KD. Inflammatory indicators such as CRP/SAA/ESR were significantly elevated, and coronary artery dilation was observed, which were also consistent with the diagnosis of KD. Therefore, even if the patient manifested with shock based on infection, attention should still be paid to distinguish KDSS by the series of clinical manifestations of KD, especially in older children.

KDSS complicated with MAS is extremely rare. Currently, there is no unified diagnostic standard for KD complicated with MAS. The most widely used criteria in clinical practice are still the HLH criteria or the MAS criteria in Systemic Juvenile Idiopathic Arthritis (sJIA) (such as the HLH-2009 criteria and the 2016 sJIA-MAS criteria) (2, 8). The patient had hemophagocytic cells (both phagocytic granulocytes and platelets phagocytized, which was rarely reported) in bone marrow biopsy, together with hepatosplenomegaly and cytopenia, fever, liver dysfunction, hypofibrinogenemia, and progressively increased ferritin levels; thus, a definitive diagnosis of MAS was made in accordance with the HLH-2009 criteria (9).

Literature suggests that some children with KD may present with acute abdominal symptoms as the initial manifestation (10). A retrospective analysis of four cases of KDSS complicated by MAS revealed that three children developed different acute abdominal conditions, which were diagnosed as acute cholecystitis, acute appendicitis, and ileal perforation.

KD complicated with KDSS and MAS will result in a higher proportion of IVIG non-response and a higher proportion of coronary artery lesions (CAL). Growing evidence suggests that adding corticosteroids to primary IVIG therapy can decrease CAL development in patients predicted to be IVIG-resistant (11, 12). Once the diagnosis was established, the patient was immediately administered high-dose IVIG with pulsed methylprednisolone (30 mg/kg/day) for 3 days. However, his fever and some symptoms still reoccurred during the corticosteroid tapering, and CAL was worsening. Therefore, infliximab was given once, and methylprednisolone 2 mg/kg/day again and tapered more slowly. Eventually, the inflammation was completely terminated, and the CAL improved significantly during follow-up. Therefore, this case reminds us that for such patients, treatment should be more aggressive by adding second-line drugs such as steroid anti-inflammatory drugs to the initial IVIG treatment. If there is recurrence, other anti-inflammatory drugs such as TNF-*α* antagonist infliximab and IL-1 receptor antagonist anakinra should be added as early as possible. Only by terminating inflammation as soon as possible can we prevent further deterioration of CAL, and it may also help improve CAL during the recovery period (6).

Through this case and the literature review, we summarized the clinical features and treatment experience of children with KDSS and MAS: (1) Children with KDSS complicated by MAS are more likely to have IVIG non-response; (2) compared with typical KD, children with KDSS complicated by MAS have a higher incidence of CAL; (3) if the child presents with abdominal pain with no efficiency by antibiotics therapy, KD should be distinguished; (4) additional therapy for initial IVIG infusion is helpful to control inflammation. We hope this case may provide more experience for the diagnosis and management of KDSS and MAS.

## Data Availability

The original contributions presented in the study are included in the article/Supplementary Material; further inquiries can be directed to the corresponding author.
